# Immunogenicity and efficacy following sequential parenterally-administered doses of *Salmonella* Enteritidis COPS:FliC glycoconjugates in infant and adult mice

**DOI:** 10.1371/journal.pntd.0006522

**Published:** 2018-05-23

**Authors:** Scott M. Baliban, Brittany Curtis, Deanna Toema, Sharon M. Tennant, Myron M. Levine, Marcela F. Pasetti, Raphael Simon

**Affiliations:** 1 Center for Vaccine Development and Global Health, University of Maryland School of Medicine, Baltimore, Maryland, United States of America; 2 Department of Medicine, University of Maryland School of Medicine, Baltimore, Maryland, United States of America; 3 Department of Pediatrics, University of Maryland School of Medicine, Baltimore, Maryland, United States of America; Oxford University Clinical Research Unit Vietnam, VIET NAM

## Abstract

In sub-Saharan Africa, invasive nontyphoidal *Salmonella* (iNTS) infections with serovars *S*. Enteritidis, *S*. Typhimurium and I 4,[5],12:i:- are widespread in children < 5 years old. Development of an efficacious vaccine would provide an important public health tool to prevent iNTS disease in this population. Glycoconjugates of *S*. Enteritidis core and O-polysaccharide (COPS) coupled to the homologous serovar phase 1 flagellin protein (FliC) were previously shown to be immunogenic and protected adult mice against death following challenge with a virulent Malian *S*. Enteritidis blood isolate. This study extends these observations to immunization of mice in early life and also assesses protection with partial and full regimens. Anti-COPS and anti-FliC serum IgG titers were assessed in infant and adult mice after immunization with 1, 2 or 3 doses of *S*. Enteritidis COPS:FliC alone or co-formulated with aluminum hydroxide or monophosphoryl lipid A (MPL) adjuvants. *S*. Enteritidis COPS:FliC was immunogenic in both age groups, although the immune responses were quantitatively lower in infants. Kinetics of antibody production were similar for the native and adjuvanted formulations after three doses; conjugates formulated with MPL elicited significantly increased anti-COPS IgG titers in adult but not infant mice. Nevertheless, robust protection against *S*. Enteritidis challenge was seen for all three formulations when three doses were given either during infancy or as adults. We further found that significant protection could be achieved with two COPS:FliC doses, despite elicitation of modest serum anti-COPS IgG antibody titers. These findings guide potential immunization strategies that may be translated to develop a human pediatric iNTS vaccine for sub-Saharan Africa.

## Author summary

Non-typhoidal *Salmonella enterica* (NTS) serovars Enteritidis and Typhimurium (including monophasic variant I 4,[5],12:i:-) are significant causes of invasive bacterial disease amongst infants and toddlers in sub-Saharan Africa, and currently, there are no approved NTS vaccines. We have demonstrated previously that immunization with *S*. Enteritidis core and O-polysaccharide (COPS) conjugated to the flagellin protein (FliC) from the homologous serovar protected adult mice from fatal infection with a Malian *S*. Enteritidis blood isolate. The target population for iNTS vaccines in sub-Saharan Africa, however, are young infants. In the current study, we evaluated *S*. Enteritidis COPS:FliC vaccination during murine infancy or adulthood. We found that COPS:FliC was immunogenic in both adult and infant mice and that co-formulation with adjuvant impacted the magnitude and quality of the immune response. Despite these differences, all vaccine formulations protected against experimental challenge in both age groups. Furthermore, robust efficacy was attainable after only two COPS:FliC doses, coinciding with the appearance of COPS-specific antibodies. The results from this study suggest that *S*. Enteritidis COPS:FliC is a promising pediatric vaccine candidate for use in sub-Saharan Africa and may help inform potential immunization strategies for iNTS COPS:FliC conjugate vaccines.

## Introduction

In sub-Saharan Africa, hospital-based blood culture surveillance of febrile pediatric admissions has revealed that invasive non-typhoidal *Salmonella enterica* (iNTS) infections caused by serovars Typhimurium, I 4,[5],12:i:-, and Enteritidis are widespread among children less than 5 years of age [1, 2]. Recent studies estimate an annual incidence of 200–400 cases per 100,000 child years in some areas, accompanied by case fatality rates of 12 to 30% [3, 4]. Genomic and phenotypic analyses have revealed several unusual traits for sub-Saharan iNTS isolates including the predominance of multi-locus sequence types not typically found in North America and Europe, gene loss in a manner analogous to typhoid and paratyphoid fever serovars, and diminished inflammatory activity in cell culture and animal models [2, 5–8]. Elucidation of the reservoir of infection and the predominant modes of iNTS transmission in sub-Saharan Africa has proved elusive, hampering efforts to implement environmental control interventions. Development of an effective iNTS vaccine for sub-Saharan Africa, therefore, remains an important public health priority, and is considered epidemiologically and immunologically feasible given the predominance of only three serovars and the established efficacy of typhoid fever vaccines as a precedent [9, 10]. Although predominantly an intracellular pathogen, *Salmonella* are susceptible to antibodies during extracellular periods prior to invading host cells or following release from infected cells.

The putative role of humoral immunity in protection against iNTS disease is supported by several important observations. Among children < 5 years of age the bulk of disease burden is found in those less than 2 years old, with peak onset occurring after 5 months of age, the point at which maternally-derived placental IgG antibodies have waned [1, 11]. Results from age cross-sectional studies of invasive *S*. Typhimurium infections among infants, toddlers and pre-school children in Malawi documented a direct relationship between disease incidence and serum bactericidal activity (SBA), wherein the drop in SBA titers during the first 8 months of life mirrored the rise in iNTS infections [12, 13]. Remarkably, the decline in disease incidence by 35 months of age was directly correlated with an increase in SBA titers to those present in newborns. A positive correlation was also found between SBA titers and antibody levels against *S*. Typhimurium lipopolysaccharide (LPS), which is the surface polysaccharide in this serovar [12].

Support for the protective capacity of anti-LPS antibodies also comes from preclinical studies in animal models of iNTS infection, whereby protection has been achieved when anti-O polysaccharide (OPS) antibodies were delivered by passive transfer or induced by active immunization [14–17]. As such, there is marked interest in the development of OPS-based vaccines to prevent iNTS infections. Whereas isolated bacterial polysaccharides generally fail to induce robust and durable antibody responses in children less than 2 years old [18], covalent linkage to protein carriers has improved the immune response to these antigens, and enabled the development of carbohydrate vaccines to prevent invasive bacterial infections in human infants (e.g., for *Streptococcus pneumoniae*, *Haemophilus influenzae* type b, *Neisseria meningitidis*). We previously reported the development of a *S*. Enteritidis core-OPS (COPS) glycoconjugate that utilized the homologous serovar phase 1 flagellin (FliC) as the carrier protein and documented efficacy of this vaccine against fatal invasive infection in adult mice experimentally challenged with a *S*. Enteritidis blood isolate from a Malian child [15, 19]. While adult mouse models are advantageous for proof-of-principle studies, they may not optimally approximate the vaccine-induced immune responses in young human infants that are the target population for this vaccine. In order to assess further the immunogenicity of *S*. Enteritidis COPS:FliC in the context of early life immunization, we evaluated herein immune responses and efficacy when vaccination was initiated either during early murine life or adulthood, and when formulated with two different adjuvants. Additionally, as we previously found that the appearance of anti-FliC IgG preceded detection of IgG anti-COPS antibodies [15, 19], we also assessed whether protection could be achieved with only one or two vaccine doses rather than the full 3-dose regimen.

## Materials and methods

### Bacterial strains

The strains used in this study are detailed in S1 Table. Growth conditions for wild-type *S*. Enteritidis R11, genetic mutant R11 Δ*fliC*, and attenuated derivative CVD 1943 (Δ*guaBA* Δ*clpP* Δ*fliD*) were described previously [20].

### Deletion of *fliC* from *S*. Enteritidis R11

*S*. Enteritidis R11 *fliC*::kan was engineered by disruption of the *fliC* gene using the lambda red-mediated mutagenesis system as described previously [21, 22]. Disruption of *fliC* was confirmed by motility assay as described [20], and Western blot with an *S*. Enteritidis-specific monoclonal antibody (clone: CA6IE2) as described [23] (S1 Fig).

### Purification and characterization of *S*. Enteritidis COPS and FliC monomers

Purified COPS and FliC monomers for use as vaccine antigens for immunization and antibody measurements by enzyme-linked immunosorbent assays (ELISAs) were generated from *S*. Enteritidis reagent strain CVD 1943, as described [20, 23]. Endotoxin removal was confirmed by endpoint limulus amebocyte lysate assay with an Endosafe PTS system (Charles River, MA). Nucleic acid removal was assessed by A260 nm for COPS and Quant-IT Sybr Green assay (Life Technologies, CA) for FliC. Removal of host cell protein in the COPS preparation was confirmed with the bicinchoninic acid (BCA) assay (Thermo, MA) and by SDS-PAGE with Coomassie staining for FliC. *S*. Enteritidis COPS identity and molecular size were confirmed by Dionex HPAEC-PAD and HPLC-SEC respectively as described [16]. Flagellin identification was accomplished by SDS-PAGE/Western blot analysis with monoclonal antibody CA6IE2, and confirmation of monomeric form by HPLC-SEC as described [23].

### Synthesis of COPS:FliC conjugates

Bioconjugation of COPS to FliC was performed essentially as described [16, 19]. Briefly, *S*. Enteritidis FliC monomers were suspended to 5 mg/mL in 100 mM MES / 0.1% Tween 20, pH 6.5. Adipic acid dihydrazide (ADH) was then added to 0.5 M and the reaction brought to 5 mg/mL *N*-(3-Dimethylaminopropyl)-*N*′-ethylcarbodiimide with incubation for 12–16 h at 4°C. The ADH-derivatized FliC was then dialyzed with 10 kDa molecular weight cutoff dialysis cassettes (Thermo, MA) against 3 x 2,000 volumes of 50 mM sodium tetraborate, 300 mM sodium chloride, 0.1% Tween 20 pH 9.15, and then concentrated to 15 mg/mL with 10 kDa Amicon Spin filters (Millipore, MA). *S*. Enteritidis COPS was brought to 10 mg/mL and precooled by incubation on ice. Activation at random polysaccharide hydroxyls was achieved by addition of 150 mg/mL 1-cyano-4-dimethylaminopyridinium tetrafluoroborate (CDAP) in acetonitrile at a ratio of 0.5 mg CDAP per mg polysaccharide. The pH was raised to pH 9.5 using dimethylaminopyridine as the base and maintained using 0.5 M sodium hydroxide as needed. The reaction was incubated for 5 min on ice, and then added to an equal amount of ADH-derivatized flagellin, and mixed by tumbling rotation for 2 h at room temperature and then 18 h at 4°C, at which point the reaction was quenched with 2 M glycine pH 9. Unreacted protein and polysaccharide were removed by size-exclusion chromatography using Superdex 200 resin (GE/Amersham, NJ) with an AKTA Purifier (GE Biosciences, NJ). Fractions that contained material of ≥100 kDa (verified by SDS-PAGE) were pooled and used for subsequent immunization. Polysaccharide and protein concentrations in the final conjugate preparation were assessed by the resorcinol-sulfuric acid method and BCA assay respectively [16].

### Ethics statement

All animal studies were performed in facilities that are accredited by the Association for Assessment and Accreditation of Laboratory Animal Care and were in compliance with guidelines for animal care established by the US Department of Agriculture Animal Welfare Act, US Public Health Service policies, and US federal law. All animal experiments were in compliance with study protocols (1114008) approved by the University of Maryland School of Medicine Institutional Animal Care and Use Committee.

### Mice

Experimental CD-1 adult females and male/female breeders were purchased from Charles River Laboratories (MA). Pups were bred and raised in the animal facility at the University of Maryland, Baltimore.

### Immunization, sera collection and challenge

Male and female infant (2 weeks old) or female adult (6–8 weeks old) CD-1 mice were immunized with 5 μg FliC or *S*. Enteritidis COPS:FliC (5 μg polysaccharide per dose) delivered in a 50 μL (infants) or 100 μL (adults) volume distributed across both gastrocnemii. Adjuvanted *S*. Enteritidis COPS:FliC formulations were generated as follows: for alum adsorptions, Alhydrogel aluminum hydroxide wet gel suspension (“alum”; Brenntag, Germany) was added to sterile-filtered *S*. Enteritidis COPS:FliC at a ratio of 28.6 mg Al per mg FliC (95% adsorption), and incubated on ice for 30 min with gentle mixing. The solution was pelleted (5 min at 10,000 x g), and the supernatant discarded to remove un-adsorbed *S*. Enteritidis COPS:FliC. Formulations of *S*. Enteritidis COPS:FliC with monophosphoryl lipid A (“MPL”; InvivoGen, CA) were generated by admixture with 1 μg of MPL followed by sterile filtration. In all experiments, control mice received sterile-filtered PBS. Serum was collected from the retro-orbital plexus or facial vein at baseline (for adult mice) and 12–14 days after each immunization unless otherwise indicated. For challenge studies, mice were infected intraperitoneally with 1 x10^6^ CFU of *S*. Enteritidis R11 or R11 Δ*fliC* in 500 μL of sterile PBS. Animals were monitored daily for 14 days after infection. Weights were recorded daily, and any animal that appeared moribund (displaying lethargy or non-responsiveness, unkempt fur, hunched posture and/or ≥ 20% weight loss) was euthanized by CO_2_ inhalation and recorded as having succumbed to challenge.

### Serum antibody analyses

**(i) Antibody titer:** Anti-COPS and anti-FliC immunoglobulin were measured by ELISA, as described previously [16]. In summary, medium binding, 96-well microplates (Greiner Bio-One, NC) were coated with 5 μg/mL of *S*. Enteritidis COPS or *S*. Enteritidis FliC for 1 hour at 37°C. Plates were blocked with 10% Omniblok (AmericanBio, MA) diluted in 1x PBS and incubated at 37°C for 2–3 hours. Serum samples were diluted in blocking buffer containing 0.05% Tween-20 (Sigma, MO) on a separate non-treated, microplate (Costar, NY) and were then transferred to the blocked ELISA plates and incubated at 37°C for 1 hour. Anti-COPS and anti-FliC immunoglobulin were detected with horseradish-peroxidase (HRP)-conjugated secondary antibodies specific for mouse IgG (SeraCare, MD) or IgG1, or IgG2b (Southern Biotech, AL) diluted 1:1,000 in blocking buffer containing 0.05% Tween-20; plates were incubated at 37°C for 1 hour. Tetramethylbenzidine substrate (“TMB”, SeraCare) was added to each well and incubated at room temperature, in darkness, with mild rocking for 15 minutes. The reaction was quenched with 1M phosphoric acid, and the absorbance at 450 nm was read using a Multiskan FC microplate photometer (Thermo, MA). End-point titers were calculated by interpolation of linear regression curves from a standard 7-parameter curve generated with pooled anti-*S*. Enteritidis hyperimmune mouse sera generated from *S*. Enteritidis COPS:FliC-immunized adult CD-1 mice that had convalesced from *S*. Enteritidis R11 challenge (University of Maryland, Baltimore). Titers were reported as the inverse dilution at which an OD_450_ of 0.2 over background was observed and represented in ELISA units (EU)/mL.

**(ii) Avidity:** Changes in anti-COPS and anti-FliC IgG avidity were assessed with urea as a chaotropic agent. For these analyses, the ELISA protocol described above was followed with the following exceptions: before the addition of secondary antibody, plates were washed three times and 200 μL of either 6M urea (Sigma, MO) or PBS were added to each well. Plates were incubated at room temperature with mild rocking for 10 minutes before washing another three times followed by addition of secondary antibody. Antibody titer in the presence or absence of urea was calculated as above. The avidity index was determined as follows: (IgG titer with 6M urea treatment / IgG titer with PBS) x 100. Values that were >100% were adjusted to 100%.

### Statistical analyses

Statistical analyses were performed using GraphPad Prism v6 (GraphPad Software, CA), and no data points were excluded from analysis. For ELISA analyses, the majority of data did not meet the criteria for Gaussian distribution, and as such, statistical comparisons were accomplished using the non-parametric Mann-Whitney U test (two-tailed, α = 0.05). Adjustments for multiple comparisons were not made. Survival curves of challenged mice were compared by the log-rank test, and mean time till death (MTTD) for each group was calculated by averaging the days at which challenged mice succumbed to infection. Vaccine efficacy was calculated based off of the attack rate (AR) in control and vaccinated mice as follows: (AR_controls_−AR_vaccinated_)/AR_controls_) x 100. A two-tailed Fisher’s exact test (FET, α = 0.05) was utilized to compare the survival proportions between vaccinated mice and controls. P-values of ≤ 0.05 were considered statistically significant.

## Results

### *S*. Enteritidis COPS:FliC immunogenicity in infant and adult mice

**Fig 1:**
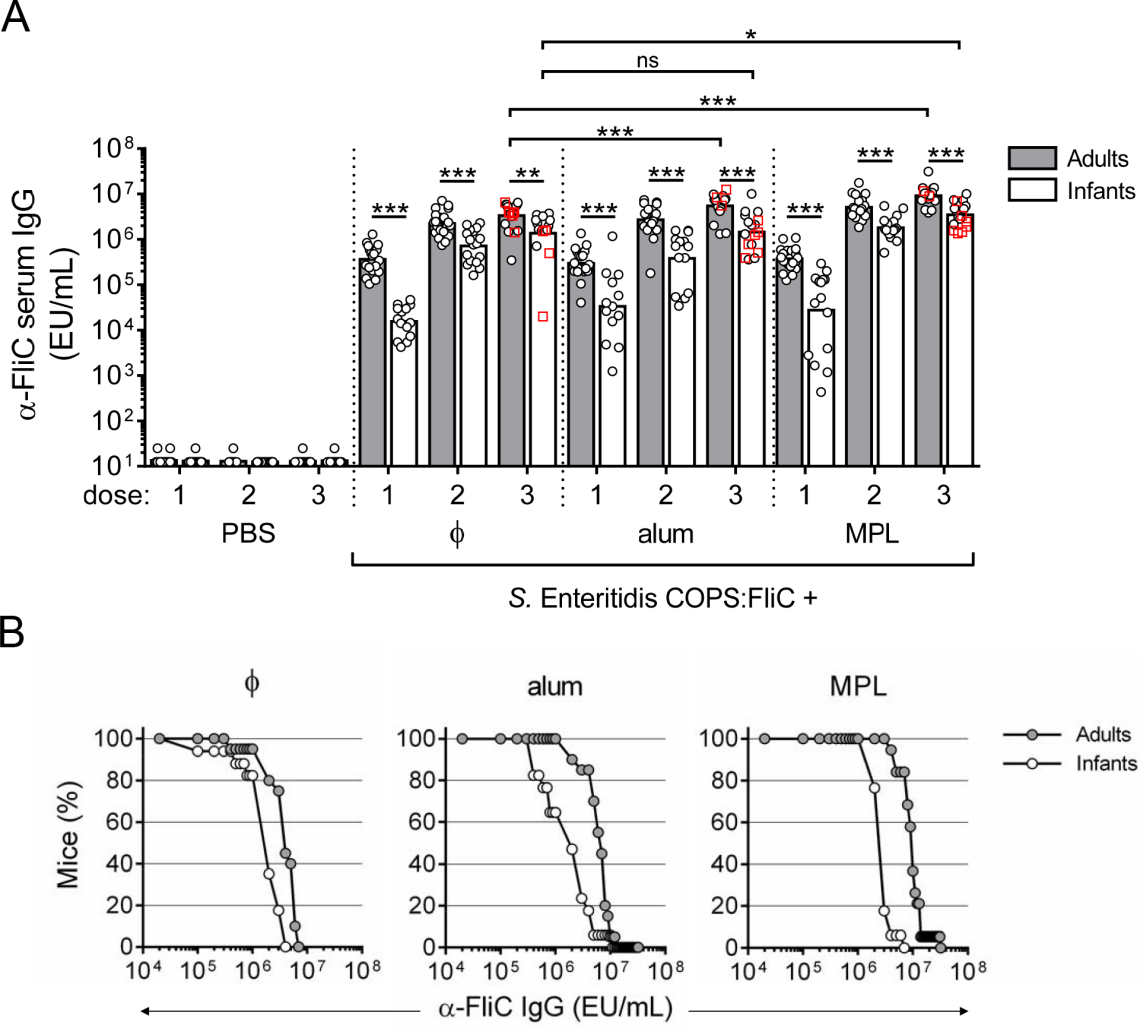
Anti-FliC IgG responses in adult and infant mice after immunization with *S*. Enteritidis COPS:FliC alone or formulated with different adjuvants. Infant and adult mice (*n* = 16–20/group) were immunized with PBS or *S*. Enteritidis COPS:FliC formulated alone (ɸ), adsorbed to alum, or admixed with MPL. (A) Serum anti-FliC IgG titers taken 12–14 days after each dose were determined by ELISA. Each point represents an individual mouse. Red squares indicate mice that succumbed to subsequent challenge. Bars represent the GMT for adults (grey) and infants (white), and were compared using a two-tailed Mann-Whitney U test. Adjustments for multiple comparisons were not made. ns, not significant. *P ≤ 0.05; **P ≤ 0.005; ***P ≤ 0.0005 for indicated comparisons. (B) Reverse cumulative distribution curves for post 3^rd^ immunization anti-FliC IgG titers for adults (grey circles) and infants (white circles) are depicted.

We previously reported that unadjuvanted *S*. Enteritidis COPS:FliC conjugates were immunogenic in adult mice, producing robust titers of anti-FliC IgG after one dose, whereas the highest anti-COPS IgG titers appeared after three immunizations [15, 19]. The immunological maturity of IgG antibody responses in 7–28 day old mice has been shown to approximate that of human infants, whereupon transition to an adult immune system occurs after approximately 28–35 days [24]. In order to compare immune responses to *S*. Enteritidis COPS:FliC in the context of an immature immune system and when formulated with an adjuvant, infant (2-week old) or adult (6–8 week old) mice were vaccinated with 3 intramuscular doses, administered at 2-week intervals, of either PBS or 5 μg *S*. Enteritidis COPS:FliC formulated alone or with alum or MPL adjuvant. *S*. Enteritidis COPS:FliC delivered alone or in the presence of either adjuvant was well-tolerated. Control mice administered PBS alone did not exhibit detectable levels of IgG antibody for either antigen throughout the experiment. Overall, the kinetics of appearance of serum anti-FliC IgG were similar between *S*. Enteritidis COPS:FliC alone and the different adjuvanted formulations. After the 1^st^ immunization with *S*. Enteritidis COPS:FliC-containing formulations, all adult mice seroconverted to anti-FliC IgG with similar geometric mean titers [GMTs] (Fig 1A). Similarly, 100% of the infant mice immunized with *S*. Enteritidis COPS:FliC manifested measurable anti-FliC IgG after the priming dose, albeit with lower end-point titers and greater individual variation compared to adults. Anti-FliC IgG titers were essentially maximal after the 2^nd^ dose for all groups. After three doses, the anti-FliC antibody levels for infant mouse groups were ~2- to 7-fold lower than that of the adults, but nevertheless robust (≥ 10^5^ EU/mL) and comparable among all animals within each experimental group (Fig 1B).

**Fig 2:**
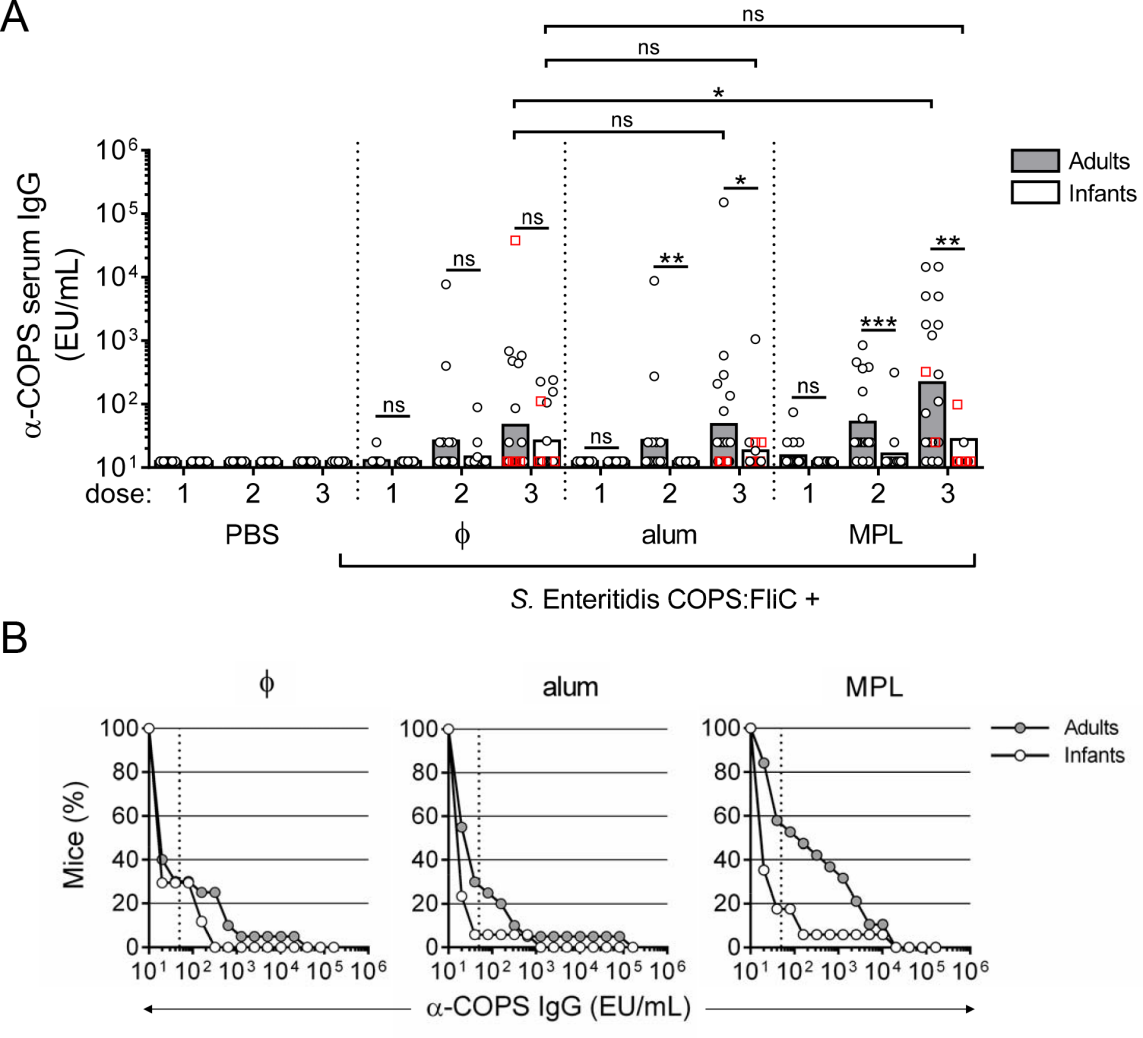
Anti-COPS IgG responses in adult and infant mice after immunization with *S*. Enteritidis COPS:FliC alone or formulated with different adjuvants. (A) Infant and adult mice (*n* = 16–20/group) were immunized as described in Fig 1. Serum anti-COPS IgG titers taken 12–14 days after each dose were determined by ELISA. Each point represents an individual mouse. Red squares indicate mice that succumbed to subsequent challenge. Bars represent the GMT for adults (grey) and infants (white), and were compared using a two-tailed Mann-Whitney U test. Adjustments for multiple comparisons were not made. ns, not significant. *P ≤ 0.05; **P ≤ 0.005; ***P ≤ 0.0005 for indicated comparisons. (B) Reverse cumulative distribution curves for post 3^rd^ immunization anti-COPS IgG titers for adults (grey circles) and infants (white circles) are depicted. Dotted lines indicate the cut-off for seroconversion (50 EU/mL), which represents a 4-fold rise over the anti-COPS IgG GMT for PBS controls.

With regard to anti-COPS IgG, endpoint titers and kinetics of seroconversion for both adults and infants immunized with *S*. Enteritidis COPS:FliC were lower relative to those directed against the FliC carrier protein (Fig 2A). Serum anti-COPS IgG was detectable in some infant and adult mice after the 2^nd^ immunization, with higher titers occurring after the 3^rd^ dose. Formulation with MPL significantly improved anti-COPS IgG levels after 3 doses in mice immunized as adults relative to unadjuvanted controls (*P* ≤ 0.05), with 47.4% of the adult mice receiving MPL formulated vaccine seroconverting to a titer of ≥ 50 EU/mL (4-fold over PBS) compared to 30% of mice in the unadjuvanted and alum groups (Fig 2B). By comparison, none of the adjuvant formulations enhanced the anti-COPS immune response in mice primed during infancy compared with unadjuvanted *S*. Enteritidis COPS:FliC.

### Anti-FliC IgG and anti-COPS IgG avidity and isotype

**Fig 3:**
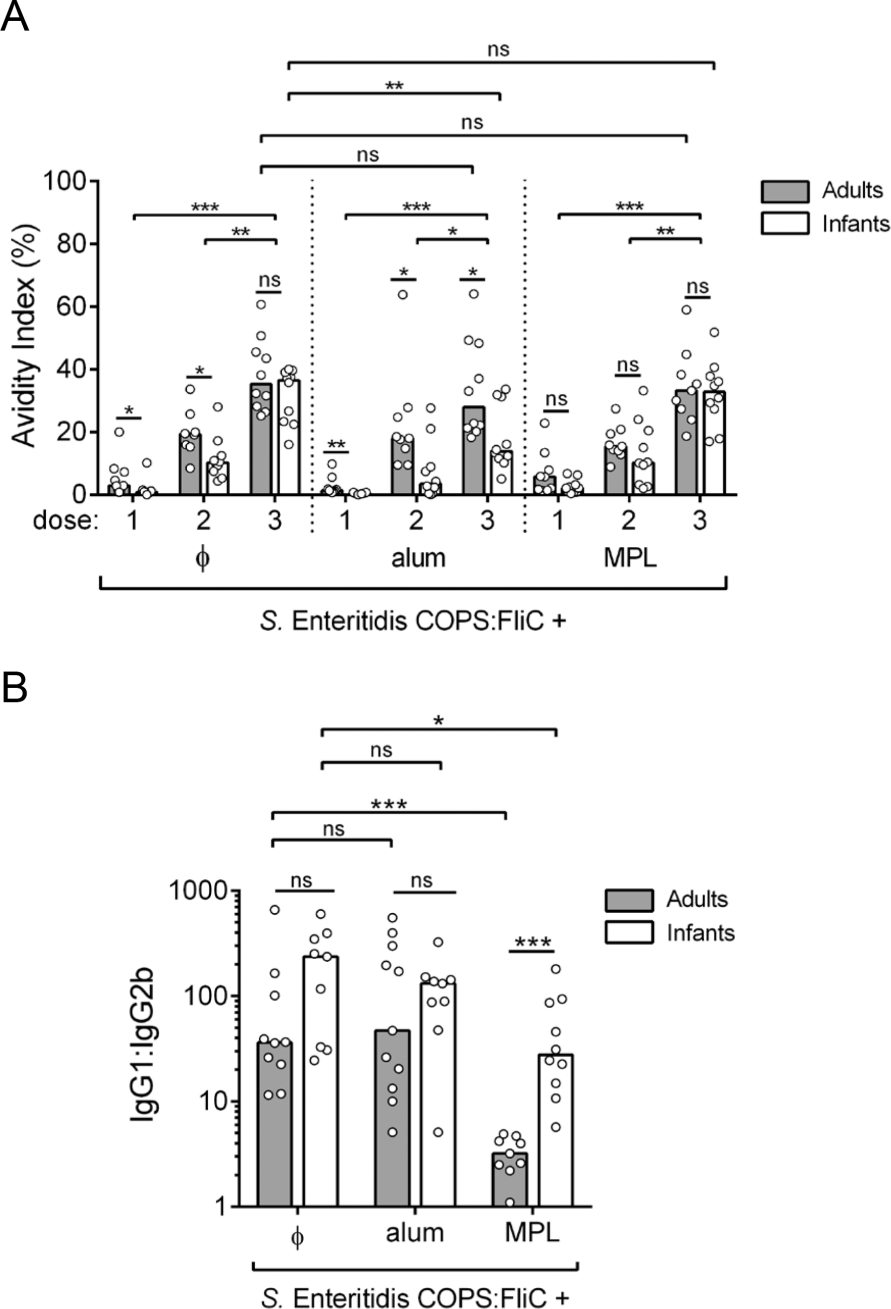
Isotype and avidity of anti-FliC IgG in adult and infant mice after immunization with *S*. Enteritidis COPS:FliC alone or formulated with different adjuvants. (A) The avidity of anti-FliC IgG in sera taken after each immunization (*n* = 8–10) was determined by sensitivity to urea treatment in an ELISA format. (B) Post 3^rd^ immunization FliC-specific serum IgG1 and IgG2b titers were determined by ELISA (*n* = 9–10). The ratio of the two titers is plotted. Each point represents an individual mouse. Bars represent the median for adults (grey) and infants (white) that was compared using a two-tailed Mann-Whitney U test. Adjustments for multiple comparisons were not made. P-values ≤ 0.05 were considered to be statistically significant; ns, not significant. *P ≤ 0.05; **P ≤ 0.005; ***P ≤ 0.0005 for indicated comparisons.

**Fig 4:**
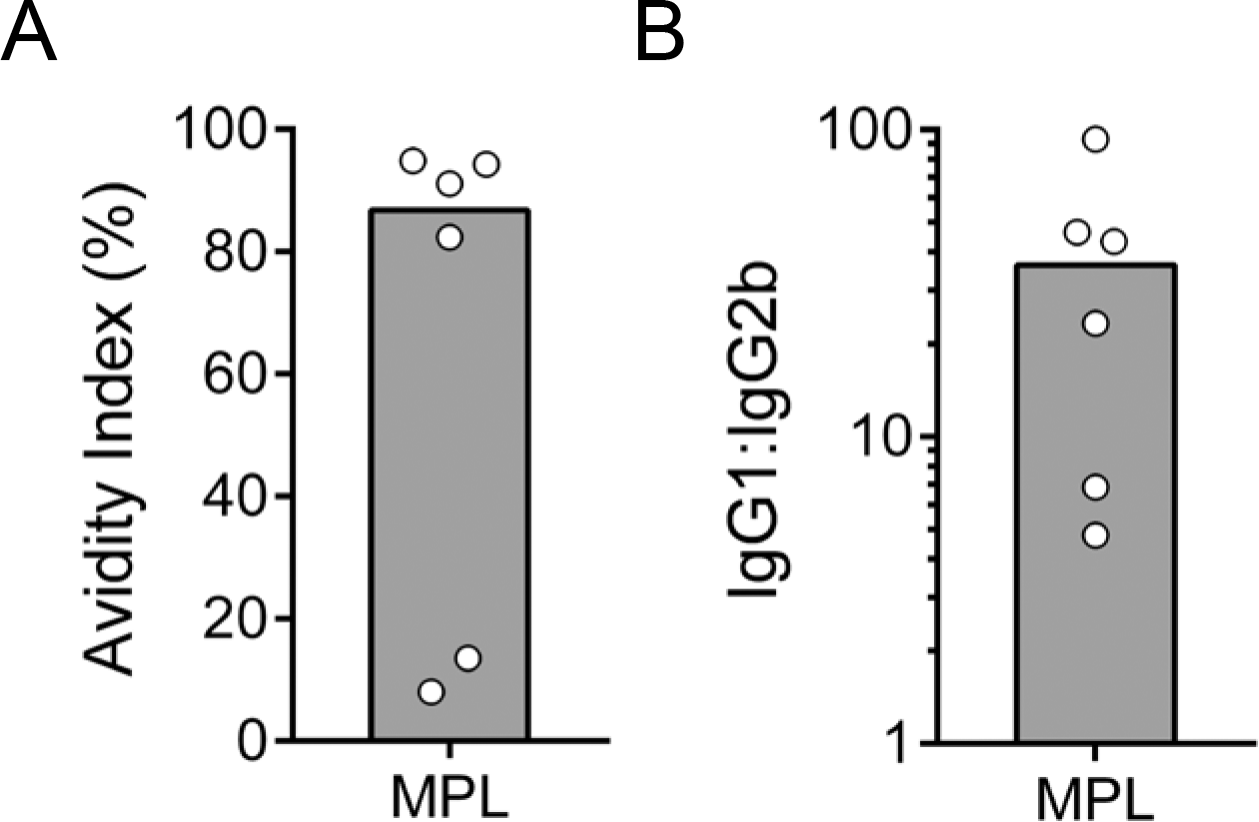
Isotype and avidity of anti-COPS IgG in adult mice after immunization with *S*. Enteritidis COPS:FliC formulated with MPL. (A) The avidity of anti-COPS IgG after the 3^rd^ immunization (*n* = 6) was determined by ELISA. (B) Post 3^rd^ immunization serum COPS-specific IgG1 and IgG2b titers were determined by ELISA. The ratio of the two titers is plotted. Each point represents an individual mouse. The bar represents the median.

To assess the quality of the serum antibody response elicited by COPS:FliC alone or formulated with an adjuvant, IgG isotype profiles and avidity indices in post-vaccination sera were evaluated. A step-wise increase in the FliC-specific IgG avidity index (AI) was observed after each immunization, and by the completion of the vaccine regimen, most vaccine groups displayed a relatively similar FliC-specific IgG AI (28.0%–38.7%) regardless of age (Fig 3A). The exception to this were infant mice immunized with alum-adsorbed *S*. Enteritidis COPS:FliC where an AI of 13.9% was found. IgG subclass analysis of FliC serum antibodies from adult- and infant-primed mice after 3 COPS:FliC doses revealed a mixed Th1/Th2 response characterized by the predominance of IgG1 relative to IgG2b (36.2–176.8-fold IgG1 bias), that was similar between mice immunized with unadjuvanted COPS:FliC or formulated with alum (Fig 3B). Adult-primed mice immunized with MPL-adjuvanted COPS:FliC manifested a more balanced Th1/Th2 profile (4-fold IgG1 bias) as evidenced by a greater contribution from the IgG2b subclass. An increase in IgG2b was also seen when MPL-adjuvanted COPS:FliC was administered to mice during infancy, which significantly decreased the IgG1:IgG2b ratio compared to the unadjuvanted vaccine but to a lesser extent than in the adult group (*P* ≤ 0.0005). Comparable analyses of anti-COPS IgG were limited by the lower IgG titers and number of seroconverting animals; only a subset of adult sera (titer ≥ 10^3^ EU/mL) obtained after the 3^rd^ dose of *S*. Enteritidis COPS:FliC formulated with MPL (Fig 4A and 4B) could be examined. These samples had a high AI (84.7%) and COPS-specific IgG was predominantly IgG1 with a wider range of variability in the ratio of IgG1:IgG2b compared to anti-FliC antibodies (Fig 4B).

### Protective efficacy of *S*. Enteritidis COPS:FliC

**Fig 5:**
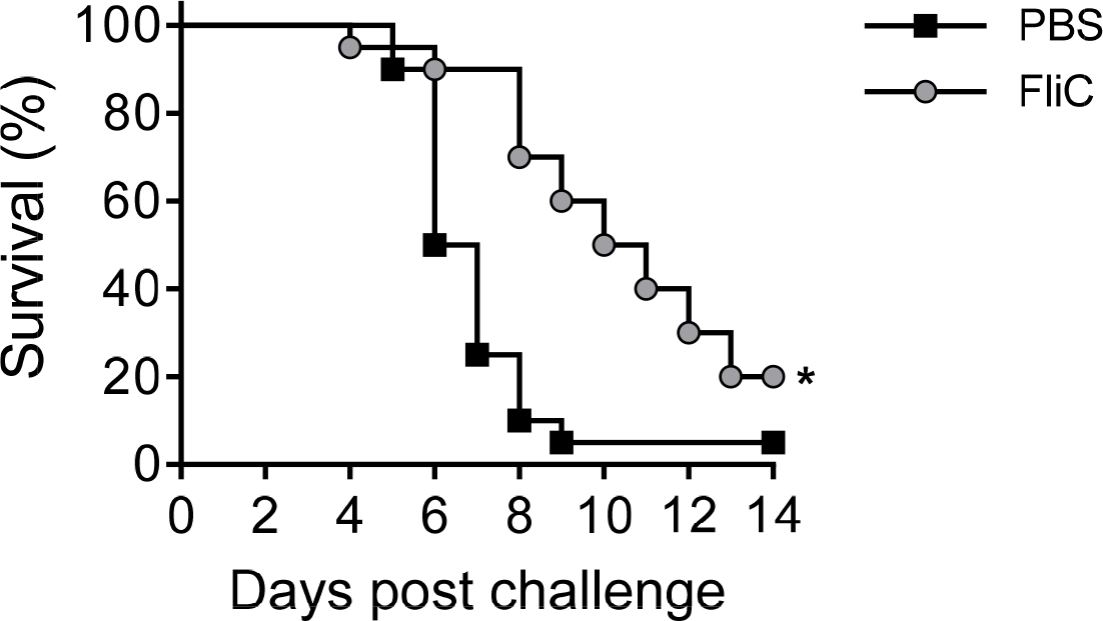
Survival of adult immunized with 3 doses of native FliC and challenged with *S*. Enteritidis R11. Kaplan-Meier survival curves for adult-primed mice immunized with 3 doses of either PBS (black squares) or FliC (grey circles) after challenge with 1x10^6^ CFU/mL of *S*. Enteritidis R11 (*n* = 20/group). Survival curves were compared using log rank analysis. For comparisons between vaccinated mice and PBS controls, P-values ≤ 0.05 were considered to be statistically significant. *P ≤ 0.0005.

**Fig 6:**
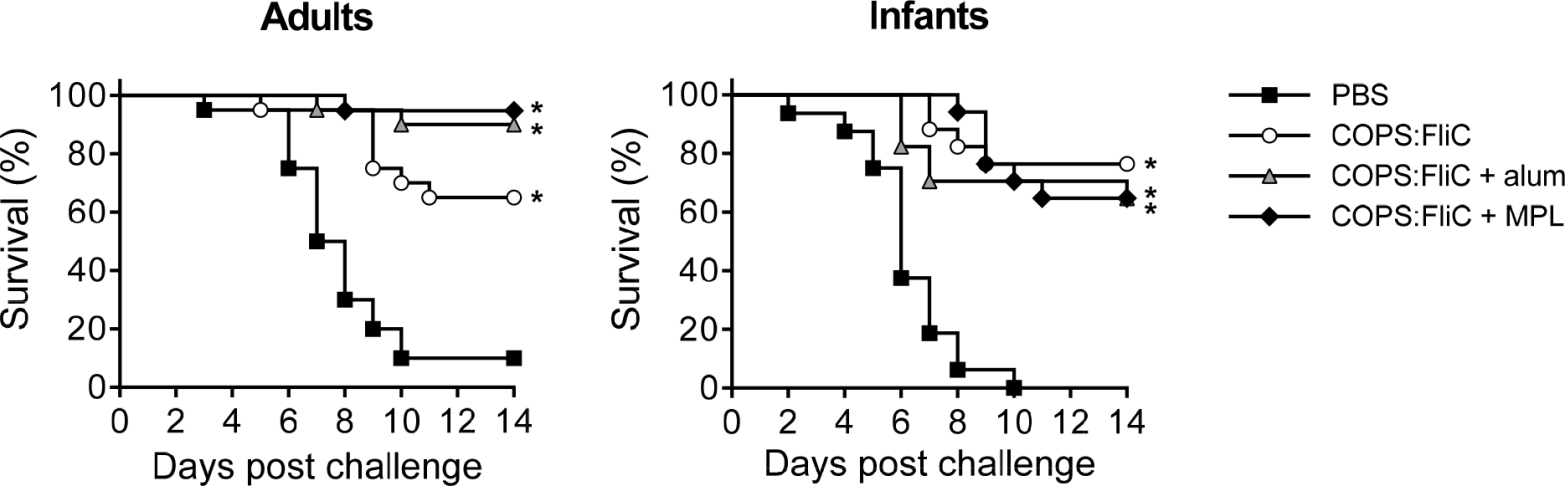
Survival of adult and infant mice immunized with 3 doses of *S*. Enteritidis COPS:FliC alone or formulated with alum or MPL and challenged with *S*. Enteritidis R11. Kaplan-Meier survival curves for adult-primed or infant-primed mice immunized with 3 doses of either PBS (black squares), unadjuvanted COPS:FliC (white circles), alum-adsorbed COPS:FliC (grey triangles), or COPS:FliC admixed with MPL (black diamonds) after challenge with 1x10^6^ CFU/mL of *S*. Enteritidis R11 *(n* = 16–20/group). Survival curves were compared using log rank analysis. Adjustments for multiple comparisons were not made. For comparisons between vaccinated mice and PBS controls, P-values ≤ 0.05 were considered to be statistically significant. *P ≤ 0.0005.

We first confirmed the protective efficacy of the FliC carrier protein against infection with *S*. Enteritidis R11, a blood isolate from Mali. Protection against mortality was negligible, but there was a significant delay in time till death (TTD) after three doses in adult mice (Fig 5). Next, we determined the protective efficacy of *S*. Enteritidis COPS:FliC against lethal infection with *S*. Enteritidis R11 in mice immunized as adults or infants with the different vaccine formulations (Fig 6). Whereas 90%–100% of PBS control mice succumbed to fatal infection, adult and infant mice immunized with three doses of *S*. Enteritidis COPS:FliC conjugate were significantly protected from death (vaccine efficacy [VE] = 61.1%–76.5%; *P* ≤ 0.0005). Amongst *S*. Enteritidis COPS:FliC-immunized adult mice, the alum and MPL formulations induced a moderate increase in VE over unadjuvanted vaccine that was significant for the MPL group (*P* = 0.04) but not for the alum group (*P* = 0.13). In infant-primed mice, however, none of the adjuvanted formulations significantly improved VE relative to *S*. Enteritidis COPS:FliC alone.

**Fig 7:**
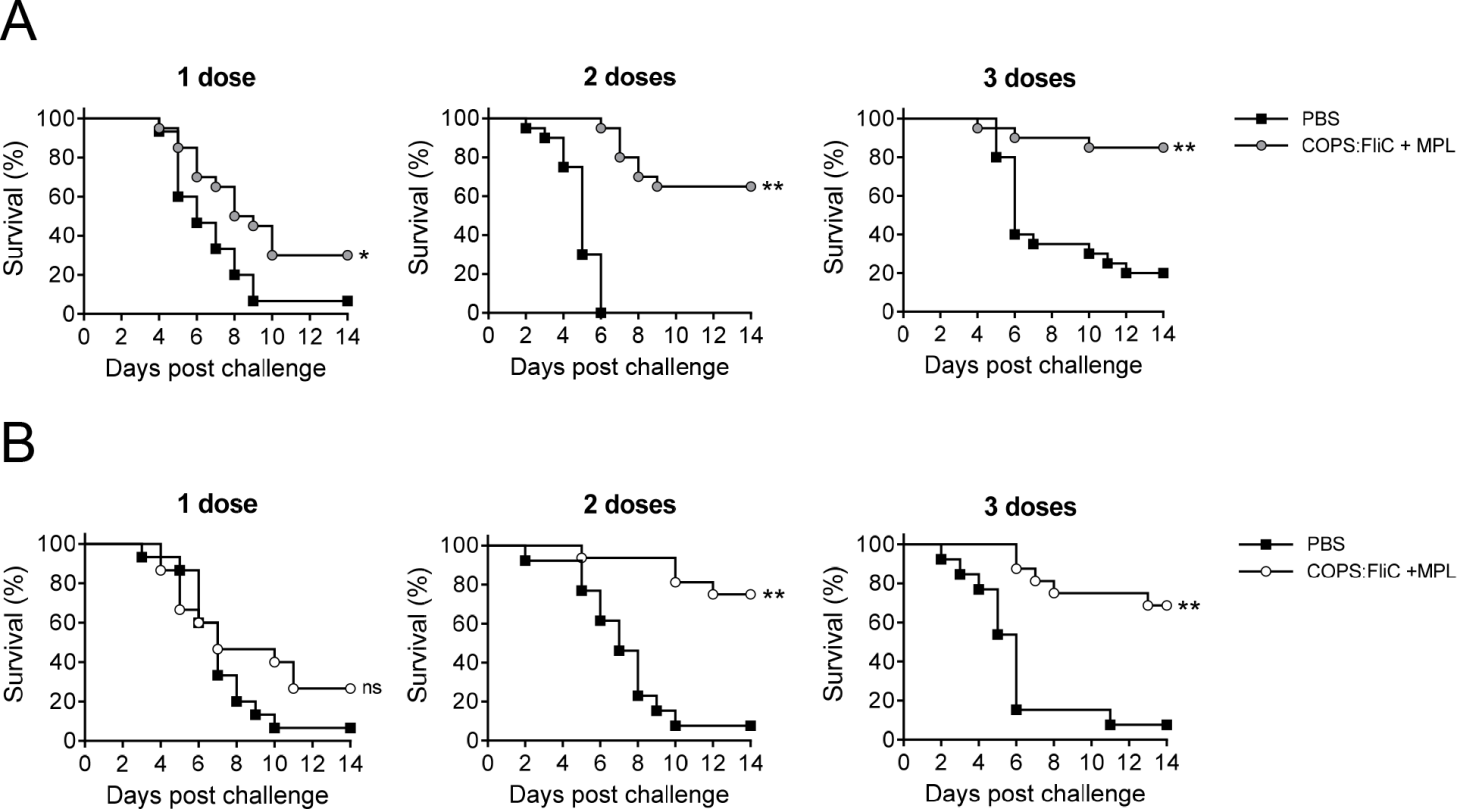
Survival for adult and infant mice immunized with 1, 2, or 3 doses of *S*. Enteritidis COPS:FliC adjuvanted with MPL and challenged with *S*. Enteritidis R11. Kaplan-Meier survival curves for adult-primed (A) or infant-primed (B) mice immunized with 1, 2, or 3 doses of PBS (black squares) or COPS:FliC (circles; grey and white color indicate adults and infants, respectively) after challenge with 1x10^6^ CFU/mL of *S*. Enteritidis R11 (*n* = 13–20/group). Survival curves were compared using log rank analysis. For comparisons between vaccinated mice and PBS controls, P-values ≤ 0.05 were considered to be statistically significant. ns, not significant; *P ≤ 0.05, **P ≤ 0.0005.

As immunization with *S*. Enteritidis COPS:FliC induced robust titers of anti-FliC IgG but negligible anti-COPS IgG after a single dose, with the highest anti-COPS IgG GMT and seroconversion rate occurring after 3 doses, we wanted to assess whether protection could be achieved with fewer doses than the full 3-dose regimen. We chose to assess the MPL-adjuvanted vaccine for these analyses as this formulation induced the highest level of anti-COPS IgG. Accordingly, infant or adult mice were immunized with 1, 2, or 3 doses of PBS or *S*. Enteritidis COPS:FliC formulated with MPL, and then challenged with *S*. Enteritidis R11 (Fig 7). We found that 2 doses of *S*. Enteritidis COPS:FliC + MPL were required to achieve significant protection from challenge (VE = 65.0%–72.9%, *P* ≤ 0.0005) and that the level of protection at this time point was similar to the that observed after 3 doses for both adult and infant mouse groups. Additionally, although measurable protection against mortality was not achieved after a single COPS:FliC + MPL immunization, there was an increase in the TTD that was significant for the adult group (*P* ≤ 0.05). Analyses of the serum anti-COPS IgM and IgG titers from these cohorts revealed a pattern similar to that seen previously where titers increased after sequential doses, with higher titers in adults compared to infant-primed groups (S2 Fig). To determine the relationship between anti-polysaccharide antibody titer and survival after challenge, we combined groups that received either 1, 2 or 3 doses into a single cohort, as this permitted assessment of a wide range of serum antibody levels. This analysis demonstrated a higher IgM and IgG anti-COPS GMT among animals that were protected versus those that succumbed (S3A Fig). Furthermore, stratifying the anti-COPS IgG response by quartiles revealed progressively higher rates of survival after challenge as IgG titer increased (S3B Fig).

**Fig 8:**
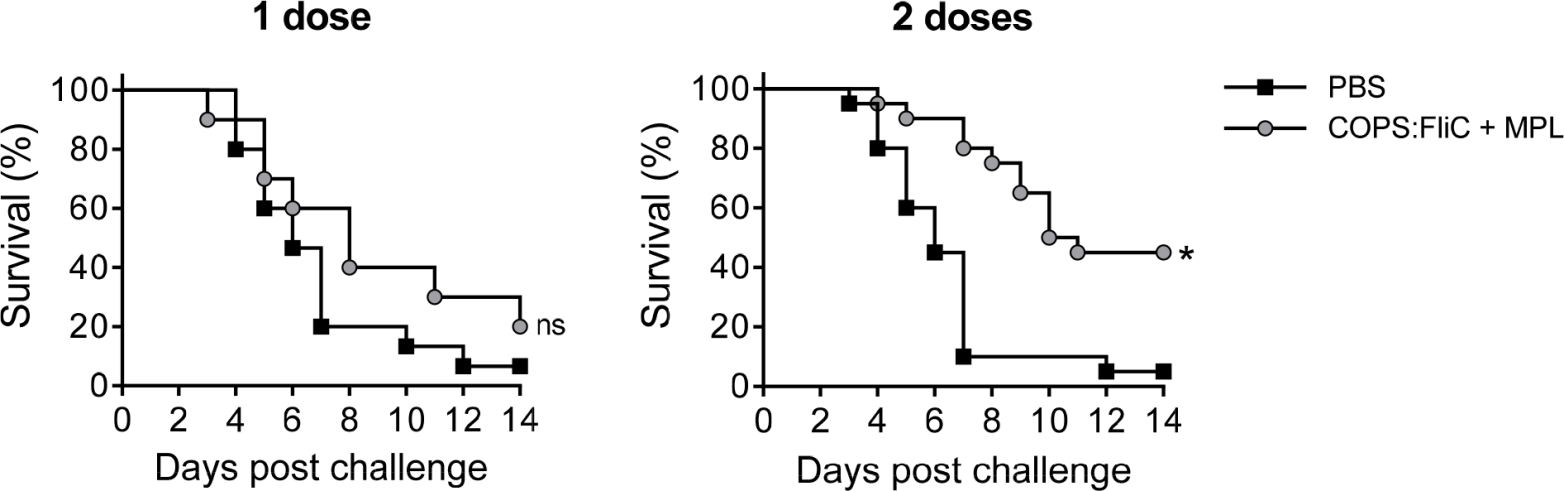
Survival for adult mice immunized with 1 or 2 doses of *S*. Enteritidis COPS:FliC adjuvanted with MPL and challenged with *S*. Enteritidis R11 *∆fliC*. Kaplan-Meier survival curves for adult mice immunized with 1 or 2 doses of PBS (black squares) or COPS:FliC (grey circles) after challenge with 1x10^6^ CFU/mL of *S*. Enteritidis R11 *∆fliC* (*n* = 10–20/group). Survival curves were compared using log rank analysis. For comparisons between vaccinated mice and PBS controls, P-values ≤ 0.05 were considered to be statistically significant. ns, not significant; *P ≤ 0.005.

In order to further dissect the contribution to protection imparted by the early FliC-specific immune response induced by the conjugate vaccine, we immunized adult mice with one or two doses of *S*. Enteritidis COPS:FliC formulated with MPL and challenged with *S*. Enteritidis R11 *∆fliC*, a phase 1 flagellin mutant (Fig 8). After receiving a single vaccine dose, the majority of vaccinated mice succumbed to challenge. Administration of two doses of *S*. Enteritidis COPS:FliC + MPL, however, afforded significant protection against *S*. Enteritidis R11 *∆fliC* challenge (VE = 47.4%, *P* ≤ 0.005), which was accompanied by a delay in TTD (*P* ≤ 0.005).

## Discussion

Analyses of the immune response of humans following natural exposure to *S*. Typhimurium in early life suggest that protection against invasive disease develops in parallel with the appearance of titers of bactericidal antibodies directed against the surface lipopolysaccharide [11, 12]. It is thus presumed that immunization with an OPS-based iNTS vaccine during early infancy may confer protection during and after the latter half of the 1^st^ year of life, the period of highest vulnerability to invasive iNTS disease among infants and toddlers living in sub-Saharan Africa [1]. As part of a program to develop a vaccine to prevent iNTS disease in human infants in Africa, we have previously documented that *S*. Enteritidis and *S*. Typhimurium COPS:FliC glycoconjugate vaccines are immunogenic and protective in adult mice [15, 16, 19]. The work described herein demonstrates that our candidate *S*. Enteritidis COPS:FliC vaccine is also immunogenic and can impart functional protection when immunization is initiated during murine infancy.

The scalable polysaccharide and flagellin purification protocols used herein generate highly pure preparations with essentially undetectable levels of residual host protein, endotoxin and nucleic acid [16, 23]. Additionally, we have documented that conjugation to polysaccharides ablates the propensity of flagellin to activate TLR5 [19]. The presence of residual TLR stimulating impurities has been hypothesized to enhance the immunogenicity of some commercial polysaccharide preparations [25]. To determine whether addition of an immuno-stimulatory adjuvant would enhance vaccine-induced immune responses to *S*. Enteritidis COPS:FliC in mice, we assessed co-formulation with different compounds that mediate adjuvanticity by different mechanisms. This included aluminum hydroxide, a known activator of the inflammasome that has a long track record of safety in human pediatric vaccines [26], or chemically-detoxified Re LPS from *Salmonella* Minnesota (MPL), a TLR4 agonist that is used as part of the adjuvant formulation in several licensed adult vaccines [27] as well as for 6 to 12-week old children receiving the RTS,S malaria vaccine [28].

In both infant and adult mice, we demonstrated 100% seroconversion and a similar prime-boost antibody response to the FliC carrier after immunization with *S*. Enteritidis COPS:FliC. Consistent with prior observations in adult mice, significant rises of anti-COPS IgG in infant or adult mice were generally apparent only after the 2^nd^ or 3^rd^ dose with more than one non-responder (by ELISA) in each group [19]. Formulation with alum did not have a measurable effect on anti-COPS or anti-FliC IgG levels or isotype profiles. Unexpectedly, we found that MPL enhanced the anti-COPS immune response in adult mice and altered the Th1/Th2 profile for anti-FliC IgG favoring the production of IgG2b in adult and infant-primed mice. While anti-COPS IgG avidity analyses were limited due to the lower number of responding animals, we did note robust avidity for anti-COPS antibodies in adult mice that received three doses of the MPL formulation. The reasons for the modest and variable anti-COPS responses observed in this study remain unknown. However, our results are similar to those reported for *S*. Enteritidis COPS conjugates with CRM_197_ which showed similarly low and variable serum post-vaccination anti-COPS IgG levels despite the use of a different carrier protein, conjugation chemistry, and mouse strain [29, 30]. One possibility for the variable antibody responses is that the antibody-antigen interactions in a portion of the polyclonal anti-polysaccharide antibody population are disrupted by the standard ELISA conditions and reagents used herein. This possibility will be addressed in future studies.

Given the differential kinetics of antibody induction by the carrier and hapten, we further assessed whether protection could be attained prior to onset of peak anti-polysaccharide immunity. Immunization with three doses of FliC alone conferred poor protection against mortality but significantly delayed the TTD in adult mice challenged with wild-type *S*. Enteritidis R11. This is consistent with prior studies where we found that passive transfer immunization in adult mice with a monoclonal IgG1 specific for *S*. Typhimurium FliC imparted partial protection against mortality and a significant increase in the TTD following challenge with a virulent *S*. Typhimurium Malian blood isolate [31]. Immunization with a single dose of COPS:FliC conjugate similarly did not significantly protect against mortality but produced a slight delay in TTD for mice infected with *S*. Enteritidis R11. Since immunization with unconjugated FliC alone prolonged survival but did not significantly protect against mortality (in our challenge conditions), poor induction of COPS-specific immunity may account for the lack of discernable protection by a single priming immunization with the conjugate. The similarly robust level of protection seen between adult mice immunized with two COPS:FliC + MPL doses and challenged with wild-type *S*. Enteritidis R11 or the Δ*fliC* mutant suggests that despite the low level of anti-polysaccharide serum IgG, protection at this intermediate point may be mediated primarily by anti-COPS antibodies.

As challenge occurred during adulthood for both groups, we sought to establish a putative relationship between serum anti-polysaccharide antibody levels and protection after challenge. We found that the anti-COPS IgM and IgG GMT in sera from mice immunized with COPS:FliC was higher in survivors compared to mice succumbing after *S*. Enteritidis challenge. Additionally, acquisition of successively higher serum anti-COPS IgG levels was associated with a proportionate drop in mortality post challenge whereby a COPS-specific IgG GMT >200 EU/mL was associated with mortality in less than 1/3^rd^ of mice. This threshold aligns with the range of the anti-COPS IgG GMT in infants and adults after two doses of COPS:FliC where we found 25–35% mortality after challenge. These results are also similar to our prior findings where COPS IgG induced by COPS:CRM_197_ vaccination in adult mice was associated with protection against lethal *S*. Typhimurium infection [16]. In this as well as the previous study, several outliers were noted wherein some animals with high anti-COPS IgG levels succumbed while others with low or undetectable titers survived. This could be reconciled by several possibilities. As discussed above, it is possible that some anti-COPS antibodies are not detected with our ELISA method. Additionally, the genetic variability in the outbred CD-1 mice used herein may also influence susceptibility to challenge and the associated protective antibody threshold among vaccinated animals with similar IgG titers. As determination of vaccine efficacy relies on group responses (i.e., measuring the proportionate reduction in the attack rate for the vaccine group versus controls), comparison of anti-COPS GMTs with proportionate survival allows for a better analysis of a possible association between vaccine-induced antibodies and protection despite the presence of outlier individual responses. Confirmation of these findings by passive transfer with vaccine-induced sera should be conducted in future studies.

The FliC-specific IgG GMT in immunized infant mice was lower than in adult mice. Nonetheless, infant anti-FliC IgG displayed an adult-like avidity and Th1/Th2 profile after three immunizations. In both adult and infant mice, the avidity of anti-FliC IgG increased sequentially after each immunization in a manner that was not directly related to the fold change in titer. These data suggest that the magnitude and avidity of serum anti-FliC IgG may not be linearly associated. Changes in avidity may reflect either a B cell intrinsic factor (e.g., affinity maturation, or the nature and diversity of the paratope repertoire), or the influence of T cell subsets important for immune maturation. Our findings for the reduced antibody responses to COPS:FliC in infant CD-1 mice can be reconciled by several possible explanations, related to the immunomodulatory effects of the early life immune system [24, 32]. Studies in neonatal mice (0–7 days old) have demonstrated that a combination of decreased co-stimulatory molecule expression coupled with immature interactions between dendritic cells, T cells and B cells may act in concert to delay the induction and limit the magnitude of germinal center responses [32]. This could result in suboptimal class switch recombination, plasma cell formation and a reduction in IgG responses to T-dependent antigens, such as glycoconjugate vaccines. It is therefore possible that the reduced anti-FliC and anti-COPS humoral immunity observed in infant mice (7–28 days old) may reflect suboptimal priming of naïve B cells or incomplete germinal center formation and feeble B cell memory. Furthermore, differences in the frequency of *S*. Enteritidis FliC- or COPS-specific B cells within the primary repertoire of infant and adult CD-1 mice may offer another possible explanation for the diminished prime-boost effect observed in infant mice. With glycoconjugate vaccines, helper T cells induced by the carrier protein are thought to promote hapten-specific B cell responses. Using a monovalent pneumococcal conjugate vaccine (Pnc1-TT) in mice, Jakobsen *et al*. demonstrated that polysaccharide-specific antibody responses increased with age, and that a weak and skewed carrier-specific T cell response generated after immunization of neonates or infants correlated with reductions in anti-polysaccharide IgG titers and protection against pneumococcal infection [33]. While COPS:FliC-induced cell-mediated immunity was not measured in this study, we speculate that the FliC-specific T cell response generated after infant priming, which may display differences in magnitude and/or breadth with respect to adult mice, contributes to the modest COPS-specific antibody response in infant mice.

Antibody responses to bacterial polysaccharides are generally limited in human infants where covalent linkage of polysaccharides to a protein carrier typically improves their immunogenicity. One proposed mechanism accounting for the poor responses of rodent and human infants to isolated polysaccharides is the lack of splenic marginal zone (MZ) B cells, which appear in the first 2–3 weeks and 2 years of life in rodents and humans, respectively [34], and are thought to be important components of the immune response to polysaccharides [18]. Interestingly, in adult mice, TLR4 stimulation has been shown to selectively promote MZ B cell proliferative responses and plasma cell differentiation [35]. By contrast, B cells from murine neonates secrete anti-inflammatory cytokines (e.g., IL-10) in response to TLR4 ligands and can suppress dendritic cell production of IL-12, a pro-inflammatory and Th1-promoting cytokine. This phenomenon is weakly evident, however, in B cells isolated from 6–8 week-old adult mice [36]. In the present study, considering that the first immunization occurred during early murine infancy (i.e., 2 weeks of age), it is conceivable that either the absence of MZ B cells or an anti-inflammatory cytokine milieu created by MPL-activated B cells at the time of priming could account for the differential adjuvant response relative to that seen in adults. We found similarly that the 3^rd^ immunization with *S*. Enteritidis COPS:FliC with MPL, delivered during adulthood (i.e., 6–8 weeks of age), was unable to drive adult equivalent anti-FliC IgG2b production in the infant-prime group. The bias toward IgG1 among vaccinated murine infants may indicate the presence of IgG1^+^ memory B cells and/or Th2-commited memory CD4^+^ T cells established during infant priming and preferentially expanded after subsequent immunizations. The importance of individual FliC-specific IgG subclasses, or combinations thereof, to clearance of *Salmonella in vivo* is not well-understood however. Using recombinant humanized IgG specific for a peptide-tagged FliC, Goh *et al*. demonstrated that IgG3 was superior at mediating *in-vitro* OPA of *S*. Typhimurium, although all IgG subclasses were opsonic to varying degrees [37].

Our findings demonstrate that *S*. Enteritidis COPS:FliC can induce protective antibody responses in mice when immunization begins during infancy. An important caveat to our immunization model is that while adult mice were immunized throughout adulthood, the early life group received their 1^st^ dose as infants (2 weeks), the 2^nd^ dose during a theoretical transition between infancy and adulthood (4 weeks) and the final dose during adulthood (6 weeks). We opted to forego an accelerated schedule here (e.g., 3 doses at weekly intervals) as this could conceivably have dampened seroconversion and the magnitude of antibody responses [38–40]. Furthermore, as protection against challenge was assessed 28 days after the final dose in order to permit maturation of adaptive immunity and resolution of the innate immune response, this further precluded assessment of vaccine efficacy during infancy. Future studies should address the role of anti-COPS antibodies in early life protection of neonates and young infants (≤14 days old) and may be accomplished by passive administration of COPS:FliC-induced antibodies. The observation that measurable protection can be achieved after only 2 doses, could also inform possible analogous vaccine schedule strategies in infants. Delaying the 2^nd^ booster dose in a 3-dose regimen (2+1) may in fact be preferable as this could potentially improve the anti-polysaccharide immune response, as has been observed in European infants receiving the pneumococcal conjugate vaccine [41]. In order to assess protection in the context of maximally induced anti-COPS IgG, we utilized COPS:FliC formulated with MPL as this produced higher anti-COPS responses in adult mice relative to the unadjuvanted formulation. It is unknown whether formulation with an adjuvant would be required for induction of immune responses to COPS:FliC in humans as immune responses to vaccination may be different relative to those obtained in rodents. Taken together, these preclinical results provide helpful insight with respect to planned first-in-human phase 1 clinical studies as well as potential immunization strategies for human infants with iNTS COPS:FliC glycoconjugates that would occur later in clinical development.

## Supporting information

S1 Fig Confirmation of *fliC* deletion in *S*. Enteritidis R11. (A) Zones of motility for *S*. Enteritidis R11 and *S*. Enteritidis R11 *∆fliC* that were stab-inoculated from overnight cultures into motility agar. (B) Western blot of crude lysates from overnight cultures of *S*. Enteritidis R11 and *S*. Enteritidis R11 *∆fliC*. Whole cell lysate (2.5 x 10^7^ CFU/lane) were separated by SDS-PAGE, transferred to a PVDF membrane and probed by Western blot analysis with a monoclonal antibody (CA6IE2) specific for *S*. Enteritidis FliC.
(TIF)

S2 Fig Sum of serum anti-COPS IgG and IgM titers for individual sera induced after 1, 2, or 3 immunizations with *S*. Enteritidis COPS:FliC formulated with MPL. Titers for serum COPS-specific IgM (black bars) and IgG (white bars) were determined in mice immunized (as described in Fig 7) as either adults (A) or infants (B) after receiving 1, 2, or 3 doses of COPS:FliC (*n* = 13–20/group). The IgG and IgM titers for a given serum sample are represented as stacked bars. Sera associated with mice that later succumbed to infection after challenge are indicated with an asterisk.
(TIF)

S3 Fig Total serum anti-COPS antibody responses after 1, 2, or 3 immunizations of *S*. Enteritidis COPS:FliC separated by survival status. (A) Titers for serum COPS-specific IgM (black bars) and IgG (white bars) induced after 1, 2, or 3 doses in adult (grey circles) and infant (white circles) mice (described in S2 Fig) were grouped into single cohorts and striated based on survival status after lethal challenge with *S*. Enteritidis R11 *(n* = 62 and 42 for alive and dead, respectively). Each point represents an individual mouse. Bars indicate the GMT, and titers were compared using a two-tailed Mann-Whitney U test. P-values ≤ 0.05 were considered to be statistically significant. *P ≤ 0.05; **P ≤ 0.005 for indicated comparisons. (B) Combined adult and infant anti-COPS IgG titers were striated by quartiles (*n* = 26/quartile) and survival ratios, defined as the number of mice that survived relative to those that succumbed to challenge, were calculated. Percent mortality for each survival ratio is given above the bar.
(TIF)

S1 Table List of *S*. Enteritidis strains used in this study. (DOCX)
