# Supplementary figures and images for: Immunogenicity and efficacy following sequential parenterally-administered doses of Salmonella Enteritidis COPS:FliC glycoconjugates in infant and adult mice
Source: PLoS Negl Trop Dis. 2018 May 23;12(5):e0006522. doi: 10.1371/journal.pntd.0006522 (PMC6002111; doi:10.1371/journal.pntd.0006522)

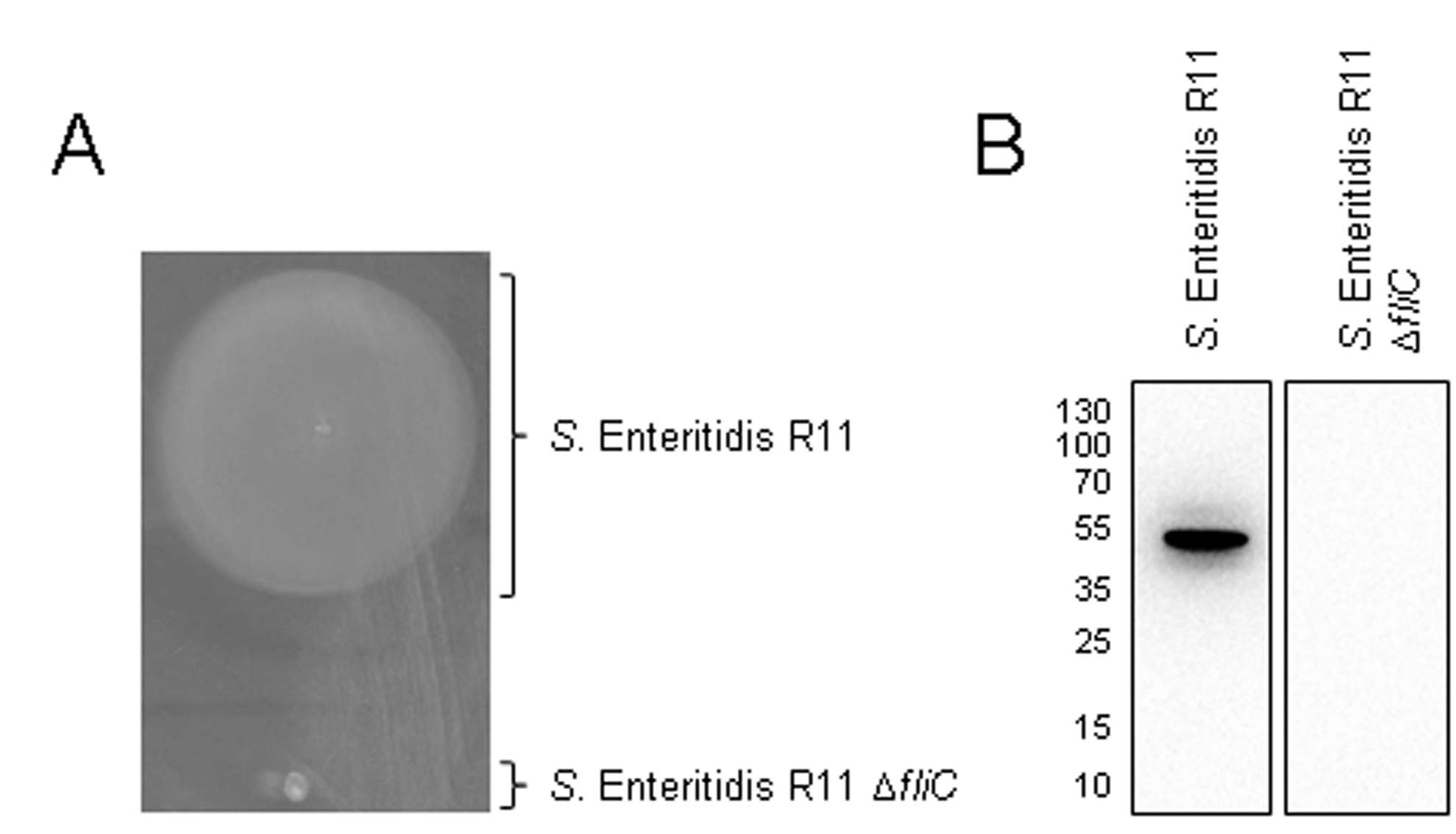

Supplement: S1 Fig — (A) Zones of motility for S. Enteritidis R11 and S. Enteritidis R11 ∆fliC that were stab-inoculated from overnight cultures into motility agar. (B) Western blot of crude lysates from overnight cultures of S. Enteritidis R11 and S. Enteritidis R11 ∆fliC. Whole cell lysate (2.5 x 10^7^ CFU/lane) were separated by SDS-PAGE, transferred to a PVDF membrane and probed by Western blot analysis with a monoclonal antibody (CA6IE2) specific for S. Enteritidis FliC. (TIF) [file pntd.0006522.s001.tif]

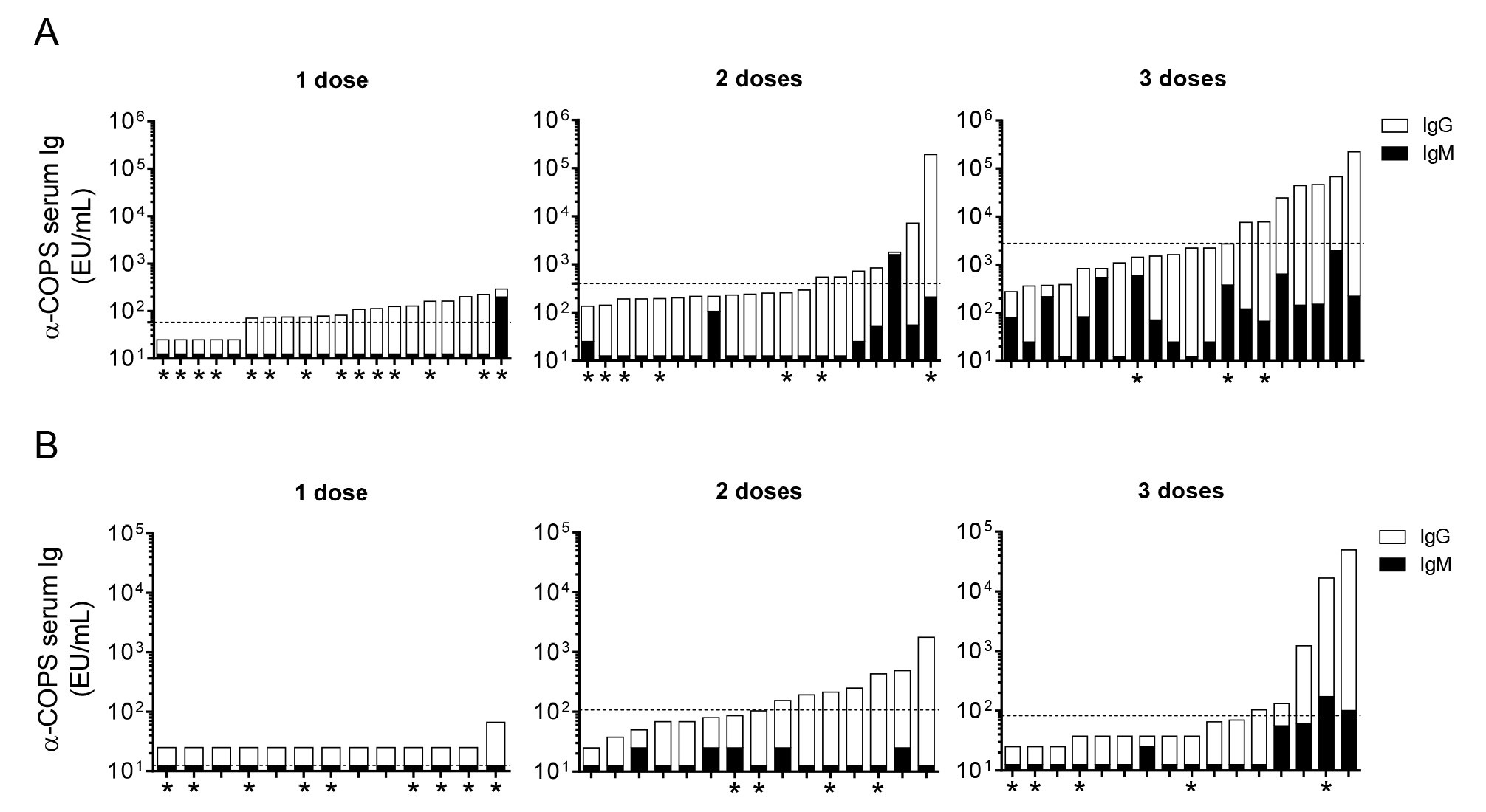

Supplement: S2 Fig — Titers for serum COPS-specific IgM (black bars) and IgG (white bars) were determined in mice immunized (as described in Fig 7) as either adults (A) or infants (B) after receiving 1, 2, or 3 doses of COPS:FliC (n = 13–20/group). The IgG and IgM titers for a given serum sample are represented as stacked bars. Sera associated with mice that later succumbed to infection after challenge are indicated with an asterisk. (TIF) [file pntd.0006522.s002.tif]

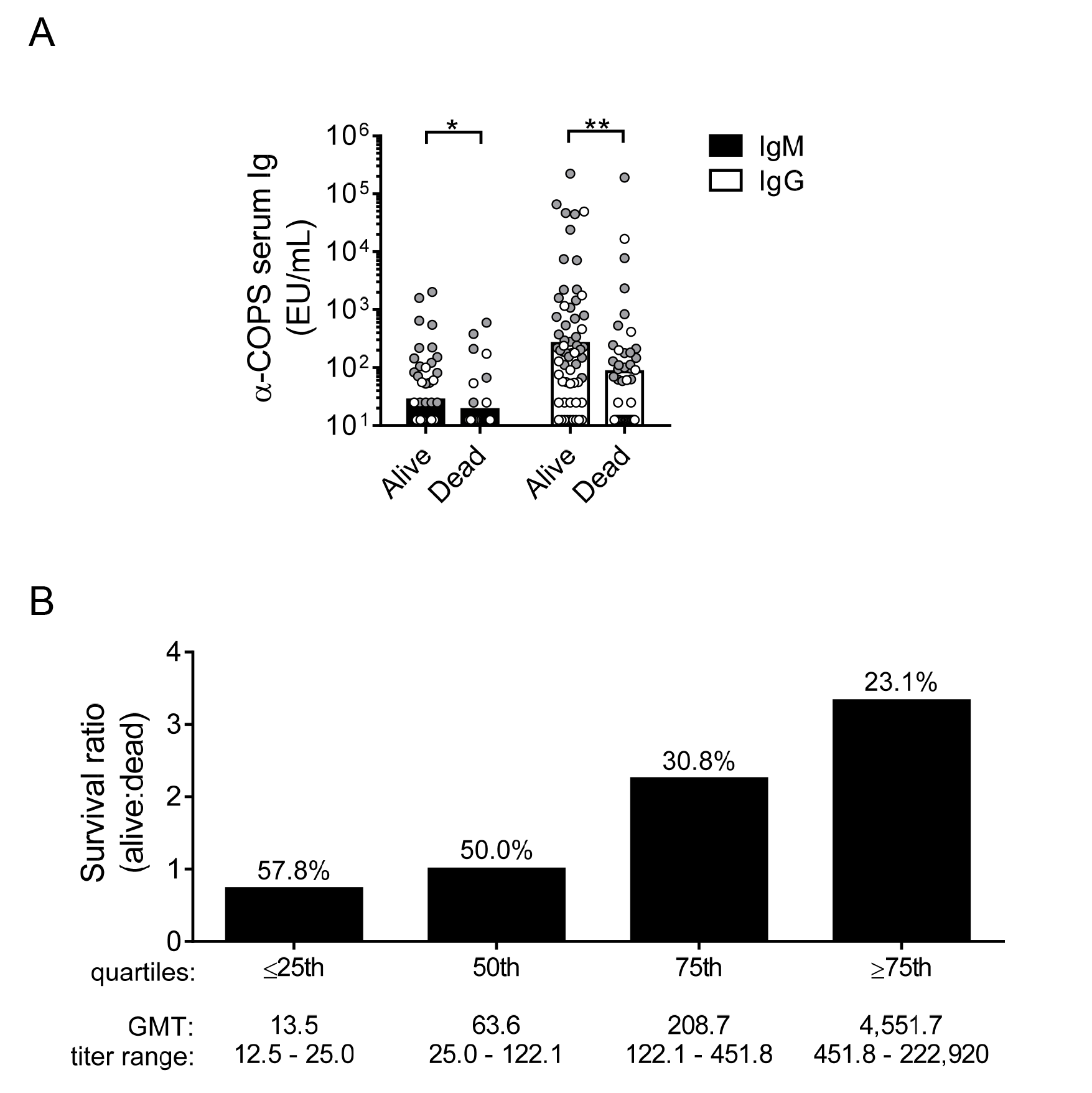

Supplement: S3 Fig — (A) Titers for serum COPS-specific IgM (black bars) and IgG (white bars) induced after 1, 2, or 3 doses in adult (grey circles) and infant (white circles) mice (described in S2 Fig) were grouped into single cohorts and striated based on survival status after lethal challenge with S. Enteritidis R11 (n = 62 and 42 for alive and dead, respectively). Each point represents an individual mouse. Bars indicate the GMT, and titers were compared using a two-tailed Mann-Whitney U test. P-values ≤ 0.05 were considered to be statistically significant. *P ≤ 0.05; **P ≤ 0.005 for indicated comparisons. (B) Combined adult and infant anti-COPS IgG titers were striated by quartiles (n = 26/quartile) and survival ratios, defined as the number of mice that survived relative to those that succumbed to challenge, were calculated. Percent mortality for each survival ratio is given above the bar. (TIF) [file pntd.0006522.s003.tif]
