# Supplementary material for: Immunogenicity and efficacy following sequential parenterally-administered doses of Salmonella Enteritidis COPS:FliC glycoconjugates in infant and adult mice
Source: PLoS Negl Trop Dis. 2018 May 23;12(5):e0006522. doi: 10.1371/journal.pntd.0006522 (PMC6002111; doi:10.1371/journal.pntd.0006522)
Supplement: S1 Table — (DOCX) [file pntd.0006522.s004.docx]

**Table S1. List of *S.* Enteritidis strains used in study.**

| ***S*. Enteritidis Strain** | **Source/characteristics** | **Reference** |
| --- | --- | --- |
| **R11** | Clinical isolate from Mali (2007) | (1) |
| **R11 *∆fliC*** | *S.* Enteritidis R11, lacks phase 1 flagellin | This study |
| **CVD 1943** | *S*. Enteritidis R11 *ΔguaBA ΔclpP* | (20) |
